# Field-deployable porcine epidemic diarrhea virus diagnostics utilizing CRISPR-Cas13a

**DOI:** 10.1080/21505594.2024.2429022

**Published:** 2024-11-19

**Authors:** Yuanyuan Wang, Dalin He, Weihua Li, Yaqin Dong, Linlin Fang, Deju Liu, Yi Tang, Shaobo Xiao

**Affiliations:** a College of Veterinary Medicine, Huazhong Agricultural University, Wuhan, China; b College of Veterinary Medicine, Shandong Agricultural University, Tai’an, China; c Department of Animal Health Standards and Regulation, China Animal Health and Epidemiology Center, Qingdao 266000, Shandong Province,China

**Keywords:** Porcine epidemic diarrhoea virus, recombinase polymerase amplification, CRISPR-Cas13a, field-deployable, diagnostics

## Abstract

Porcine epidemic diarrhoea virus (PEDV), a pathogenic microorganism that induces epidemic diarrhoea in swine, causes substantial economic damage to swine-farming nations. To prevent and control PEDV infections, the availability of upgraded and rapid virus detection techniques is crucial. The clustered regularly interspaced short palindromic repeats (CRISPR)-CRISPR-associated protein (Cas)13a system, namely, programmability of CRISPR RNA (crRNA) and “collateral” promiscuous RNase activity of Cas13a after target RNA identification. In this study, we aimed to develop a recombinase polymerase amplification (RPA)-based CRISPR-Cas13a approach for PEDV diagnosis for the first time. The results showed that up to 10 copies of the target PEDV DNA standard/µL were detected after 40 min at 37 °C. PEDV detection exhibited remarkable specificity compared to that of other selected pathogens. Additionally, this RPA-based CRISPR-Cas13a approach could be used to clinical samples, with similar performance to that of reverse transcription-quantitative polymerase chain reaction (RT – qPCR). The results of our proposed approach were visualized using either lateral flow strips or fluorescence for field-deployable viral diagnostics, thereby facilitating its use in endemic regions. Overall, our proposed approach showed good reliability, sensitivity, and specificity, suggesting that it is applicable for detecting other viruses in diagnosing diseases and inspecting food safety.

## Introduction

Since December 2010, a highly epizootic acute diarrhoeal condition presenting symptoms such as emesis, dehydration, and watery diarrhoea has been occurring in suckling piglets on multiple swine farms throughout China [1,2]. Based on these symptoms, the disease was diagnosed with porcine epidemic diarrhoea (PED) caused from PED virus (PEDV). Like *Alphacoronavirus* species from the *Coronaviridae* family, PEDV represents a positive-sense, single-stranded RNA (ssRNA) virus, with a genome length of 28 kb [3–5]. Its genome contains one long open reading frame (ORF1a, ORF1b, and ORF2–6) that encodes two 5′ large polyproteins, four 3′ structural proteins, and one auxiliary protein from ORF3 [6]. ORF1a and ORF1b generate polyproteins 1a and 1b, which can be cleaved into 16 mature non-structural proteins (nsp1–nsp16) by viral-encoded proteases [3]. ORF2 and ORF4–6 encode the structural proteins spike (S), membrane (M), nucleocapsid (N), and envelope (E) [7]. Notably, S, M, and N are targeted during epidemiological investigations, such as phylogenetic assessment, and in several serological and molecular diagnostic techniques for PEDV [8–13]. Despite their fast adaptability and high sensitivity, most existing nucleic acid assay methods require sophisticated instruments and extensive sample manipulation. In contrast, antigen-based rapid diagnostic assays do not require sophisticated instruments, but lack sensitivity and are time-consuming to develop. A desirable diagnostic approach should combine the specificity, flexibility, and sensitivity of nucleic acid testing with operational convenience and velocity of antigen-based assays. Such a method needs to be quickly developed and deployed upon incipient PEDV outbreaks. It would be appropriate for routine clinical use in underequipped laboratories or actual sites.

Programmable endonucleases exist within microbial clustered regularly interspaced short palindromic repeats (CRISPR) and CRISPR-associated (Cas) adaptive immunity systems, which are leveraged in CRISPR-assisted diagnostics [14,15]. CRISPR-Cas13a, a newly discovered CRISPR-Cas member, includes CRISPR RNA (crRNA) and Cas13a protein [16]. Reprogrammable with crRNA, Cas13a offers an approach for detecting specific RNAs [17–20]. As recently discovered in *in vitro* studies, Cas13a/crRNA is stimulated to achieve non-specific cleavage of labelled RNA after its target RNA is specifically identified, also referred to as the collateral cleavage effect [14,21]. This includes an ssRNA-linked biotin reporter or fluorophore-quencher pair, which enables visual quantification of the reaction through colorimetric or fluorescence assays. The crRNA guide zone is responsible for recognizing target RNAs via the complementary sequence, whereas activated Cas13a endonucleases collaterally cleave vicinal non-targeted RNAs [22–24]. This indicates that CRISPR-Cas13a-associated assaying of nucleic acids may overcome core viral diagnostic-related challenges.

To maximize the convenience and increase detection sensitivity, in this study, we developed an enzymatic molecular diagnostic system by integrating recombinase polymerase amplification (RPA), T7 transcription of amplified DNA to RNA, and Cas13a collateral effects. The conservative genome sequence regions of the PEDV-M protein were used for specific RPA primers and crRNA design. Their specificity was assessed using clinical samples and their sensitivity was evaluated using plasmid DNA standards. We believe that our approach has the potential to supplement reverse transcription – quantitative polymerase chain reaction (RT – qPCR) for on-site PEDV detection, which may facilitate timely surveillance.

## Materials and methods

### Viruses and clinical samples

The PEDV strain AJ1102 was isolated and stored in our laboratory according to a previous description [25]. Porcine reproductive and respiratory syndrome virus (PRRSV), transmissible gastroenteritis virus (TGEV), porcine deltacoronavirus (PDCoV), porcine parvovirus (PPV), pseudorabies virus (PRV), porcine circovirus 2 (PCV2), and swine acute diarrhoea syndrome coronavirus (SADS-CoV) were obtained from China Animal Health and Epidemiology Centre. In total, 35 clinical specimens (including faeces and intestinal samples) from piglets suspected of PEDV infection were collected from various regions of China. We cryopreserved the viruses and clinical samples at − 80 °C prior to later experimentation.

### Expression and purification of LwCas13a protein

The expression plasmid, pC013-Twinstrep-SUMO-huLwCas13a (cat. no. 90097) were obtained from Addgene. The TIANprep Rapid Mini Plasmid Kit (cat. no. DP105) from TIANGEN Biotech was used to extract plasmids, such as Cas13a protein-expressing LwCas13a, beta-lactamase-expressing AmpR, *lac* repressor-expressing lacI, and tagged protein genes (small ubiquitin-like modifier [SUMO], 6× His, and strap II). The expression and purification of LwCas13a protein were conducted as previous description [26,27]. Briefly, the LwaCas13a plasmid (50 ng) was converted into *Escherichia coli* Rosetta (DE3)-competent cells. Following this, the cells were incubated overnight inside a bioshaker in the terrific broth medium at 300 rpm and 37 °C till their optical densities ranged from 0.4 to 0.6 at 600 nm. Afterward, isopropyl-1-thio-β-D-thiogalactopyranoside (500 µM, cat. no. I6758, Sigma-Aldrich) was used to examine protein expression, followed by cultivation in a 21 °C pre-chilled bioshaker at 300 rpm and 16 °C. For cell harvesting, the culture was subjected to 10-min centrifugation at 4 °C and 5,000 rpm, resuspended in lysis buffer (500 mm NaCl, 20 mm Tris-HCl, and 1 mm dithiothreitol [DTT]; pH 7.4) containing protease and lysozyme inhibitors, and subsequently ultrasonicated (JX-650, Shanghai, China) for 25 min at the following settings: power, 200 W; duration of each sonication, 2.5 s; and duration of each pause, 10 s. After separating the lysate by centrifugation, nickel-nitrilotriacetic acid agarose was used for supernatant cultivation and an elution buffer (20 mm Tris-HCl, 250 mm imidazole, and 150 mm NaCl; pH 7.5) was used to elute the bound protein. To eliminate the aforementioned tag protein genes, LwaCas13a protein after elution was subjected to overnight digestion with SUMO protease at 4 °C, followed by purification using a heparin column (GE Healthcare Life Science). The purity of the recombinant protein was examined using sodium dodecyl sulphate-polyacrylamide gel electrophoresis. Fractions containing pure LwCas13a were dissolved in storage buffer (SB; containing 50 mm Tris-HCl, 600 mm NaCl, 2 mm DTT, and 5% glycerol; pH 7.5) and cryopreserved at − 80 °C prior to further experimentation.

### RNA extraction and complementary DNA (cDNA) synthesis

MagMAX^TM^ mirVana^TM^ Total RNA Isolation Kit (cat. no. A27828) was used to extract the viral RNAs of PEDV, PCV2, PRRSV, PRV, PPV, TGEV, and PDCoV, according to the protocols of TransGen Biotech. Subsequently, the viral RNAs were reverse-transcribed using a HiFiScript cDNA Synthesis Kit (cat. no. CW2569M; KANGWEI) and cryopreserved at − 20 °C until further use.

### PEDV-M recombinant plasmid standard synthesis

The M gene of PEDV was subjected to RT – qPCR amplification using the respective primers (Table 1). After purification using a gel extraction kit (cat. no. D2500–02; Omega Bio-tek), those polymerase chain reaction (PCR) products after amplification were cloned in Takara pMD18-T vector (cat. no. 6011; Takara), and these vectors were transfected into *E. coli* DH5α-competent cells. Sequencing of the cloned PCR fragment into the recombinant pMD18-T-M plasmid (the target PEDV DNA standard, 3038 base pair) was performed at BGI Tech (Shenzhen, China), and the sequencing results were verified. The TIANprep Mini Plasmid Kit (cat. no. DP105; TIANGEN Biotech) to acquire the plasmid, the concentration of which was estimated using a DS-11 Spectrophotometer (Denovix, USA). The copy number was determined as follows:

$$\mathrm{Copy}\;\mathrm{number}\left(\mathrm{copies}/\mu \;L\right)\;=\;\left(6.02\;\times \;10^{23}\right)\;\times \;\left(\mathrm{ng}/\mu \;L\times \;10^{-9}\right)/\left(\mathrm{DNA}\;\mathrm{length}\times \;660\right)$$

**Table 1.** Sequences of primers for target RNA, crRNA and RNA probe in this study.

Name	Sequence
PCR forward primer	ATGTCTAACGGTTCTATTCCC
PCR reverse primer	TTAGACTAAATGAAGCACTTTCT
RPA forward primer	TAATACGACTCACTATAGGGCAATCCTGAAACAGACGCGCTTCTCACTAC
RPA reverse primer	TTAGACTAAATGAAGCACTTTCTCACTATC
crRNA	GAUUUAGACUACCCCAAAAACGAAGGGGACUAAAACGACUGUGACGAAAUUAGGUAAUUGACUU
crRNA IVT template	AAGTCAATTACCTAATTTCGTCACAGTCGTTTTAGTCCCCTTCGTTTTTGGGGTAGTCTAAATCCCCTATAGTGAGTCGTATTAATTTC
T7-3 G IVT primer	GAAATTAATACGACTCACTATAGGG
RNA probe	FAM-TrUrUrUrUrUrC-BHQ1
LF-RNA reporter	FITC-rUrUrUrUrUrU-Bio

where ng/µL × 10^−9^ represents the concentration of the isolated plasmid.

### RPA primer and crRNA synthesis

RPA pre-amplification necessitates the design of corresponding primers. For maximal amplification efficiency, the preferred length scope of primers was set to 25–35 nucleotides (nt), and the preferred overall size range of amplicons was set to 80–140 base pairs [28]. Primers were prepared at melting temperatures of 54–67 °C. To identify the conserved zone, reference sequence alignment was performed for the PEDV-M gene using MegAlign and EditSeq in Lasergene 12 Core Suite (DNASTAR, lnc. Madison, WI, USA). RPA primers within the conserved nt zone of the M gene were selected. In addition, the 5’ end of the RPA forward primer was appended to a T7 RNA polymerase promoter (Table 1).

**Figure 1.**
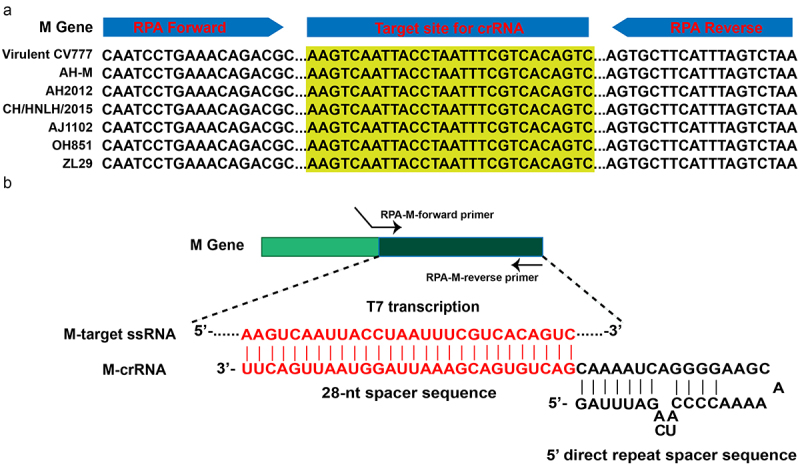
Sketch map showing the designation of clustered regularly interspaced short palindromic repeats RNA (crRNA) for porcine epidemic diarrhea virus detection. (a) The alignment of seven representative sequences for the conserved membrane gene segment. (b) Base pairing between target RNA and crRNA.

Superposition of crRNA designed for our proposed RPA-assisted CRISPR-Cas13a diagnostic strategy with RPA primers was not allowed (Figure 1a). The crRNA sequence represents the reverse complement of the transcribed ssRNA target site. Within the resultant pre-amplified RPA product, we placed a spacer sequence (28-nt long for LwaCas13a) and designed it using a web application (CRISPR-RT, http://bioinfolab.miamioh.edu/CRISPR-RT/interface/C2c2.php). Finally, we formed an integral crRNA by integrating the spacer sequence with the 5′ direct-repeat sequence (Figure 1b, Table 1). To allow T7 transcription, we appended a T7 RNA polymerase promoter upstream of the crRNA sequence such that crRNA could be produced based on DNA templates through *in vitro* transcription (IVT). Furthermore, we ordered this whole sequence as a DNA reverse complement (see Table 1 for the crRNA IVT templates).

#### Preparation and purification of crRNA

CrRNA quality is crucial to the efficiency of CRISPR-associated diagnostics. For every sample, an annealing reaction (Table S1) was performed using a standard *Taq* buffer (cat. no. B9014S; New England Biolabs, UK). Inside the PCR thermocycler, annealing of T7–3 G oligonucleotide and crRNA IVT template was accomplished as follows: 30-min at 37 °C, 5-min at 95 °C, and gradual decline at 5 °C/min to 25 °C. The annealing reactions (Table S2) were incorporated with IVT templates, following the HiScribe T7 Quick High Yield RNA Synthesis Kit instructions (cat. no. E2050S, New England Biolabs). Following a 4-h incubation at 37 °C, the reaction temperature was decreased to 4 °C inside the PCR thermocycler. RNA purification was performed using an Agencourt RNAClean XP Kit (cat. no. B9014S; Beckman Coulter), according to the manufacturer’s instructions.

### PEDV-detection using the RPA-based CRISPR-Cas13a diagnostic system

Our proposed RPA-based CRISPR-Cas13a diagnostic strategy consists of RPA and Cas13a sensing, encompassing T7 transcription. For RPA, this reaction master mix was formulated inside one 0.2-mL tube containing template (13.2 µL), dried enzyme (50 µL), rehydration buffer (29.5 µL), respective primers (each 2.4 µL, 10 µM), and distilled water. A single-pellet aliquot was added to the master mix and the reaction mixture was carefully resuspended. Following this, the entire reconstituted reaction mixture was transferred back to the original tube (0.2 mL). Following the guidelines of the TwistAmp® Basic Kit (cat. no. TABAS03KIT; TwistDx), 280 mm TwistAmp magnesium acetate (2.5 µL) was added to the reaction mixture. Next, following a 15-min tube incubation at 37 °C, fluorescence-based Cas13a sensing was conducted within a 20-µL reaction volume, including reconstituted Cas13a, crRNA, RPA pre-amplification reaction sample, MgCl_2_, recombinant nucleotide triphosphates (cat. no. N0466L) and T7 polymerase (cat. no. E2050S), RNase inhibitor (New England Biolabs, cat. no. M0314S), buffer (containing NaCl [60 mm], Tris-HCl [40 mm], and MgCl_2_ [6 mm]; pH 7.3), and reporter RNA [29]. The LightCycler (Roche Diagnostics) was used in reactions at 37 °C for 90 min, and in this process, we recorded the fluorescence values at 10-min intervals. Alternatively, the reactions can be evaluated by tracking the end products, which can be observed macroscopically under ultraviolet illumination.

**Figure 2.**
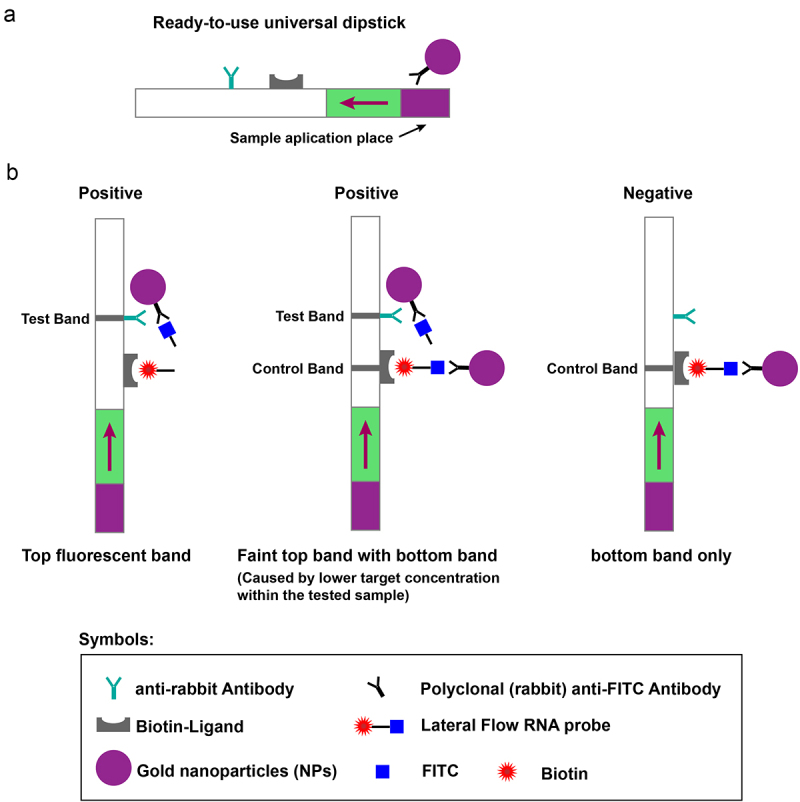
Diagrammatic illustration of the recombinase polymerase amplification-assisted clustered regularly interspaced short palindromic repeats (crispr)-crispr-associated 13a protein lateral flow sensing. The structure (a) and operating principle (b) of the lateral flow dipstick.

For Cas13a-based portable lateral flow (LF) immunosensing, an LF-RNA reporter (final dose: 1 µM) was substituted for the RNA probe in the reaction system [30], which adopted an RNA reporter flanked by biotin and fluorescein isothiocyanate (FITC) at either end (Table 1). A biotin – ligand series bound to biotin on the lateral flow dipstick, capturing the entire intact probe. Gold nanoparticle (NP)-conjugated anti-FITC antibodies were bound to the reporter FITC end, which developed into a dark purple colour at the bottom of the dipstick band (Figure 2a). Upon cleavage of the RNA reporters due to target existence and collateral effects, the NP-conjugated antibodies migrated to the top band, suggesting the existence of the target (Figure 2b). Inside an incubator, 1 h of lateral-flow Cas13a sensing was performed at 37 °C. This whole reaction mixture was subjected to a 5-min incubation using HybriDetect Assay Buffer (80 µL), as described with the utilization of com mercial detection strips following the protocols of the manufacturer (Twist-Dx, cat. no. MILENIA01). A phone camera was used to photograph the dipstick.

### Specificity and sensitivity of our rpa-based CRISPR-Cas13a diagnostic system

To assess specificity, our RPA-based CRISPR-Cas13a diagnosis strategy was applied to seven different viruses (PDCoV, PRRSV, SADS-CoV, TGEV, PRV, PPV, and PCV2) and a negative control (NC) sample (nuclease-free water). To assess sensitivity, the template was subjected to 10-fold gradient dilutions (10^7^–10° copies/µL) of the pMD18-T-M plasmid standard using the EASY Dilution (cat. no. 9160; Takara) in the reaction system (20 µL). Nuclease-free water served as the NC.

### Application in clinical samples

To assess the reliability of our proposed RPA-based CRISPR-Cas13a diagnostic system, 35 clinical field samples collected from suspected PEDV-infected piglets were subjected to RT – qPCR [31] and subsequent RPA-assisted CRISPR-Cas13a diagnosis.

### Statistical analyses

Prism 5.0 (GraphPad, San Diego, CA, USA) was used for data analysis. Data are represented as the mean ±standard error of the mean. For inter-assay differences, analysis of variance was performed to assess the data using Bonferroni correction (SPSS 17.0, IBM, USA). Statistical significance was evaluated using a *p*-value cut-off of 0.05.

## Results

### Process of the rpa-based CRISPR-Cas13a diagnostic system

**Figure 3.**
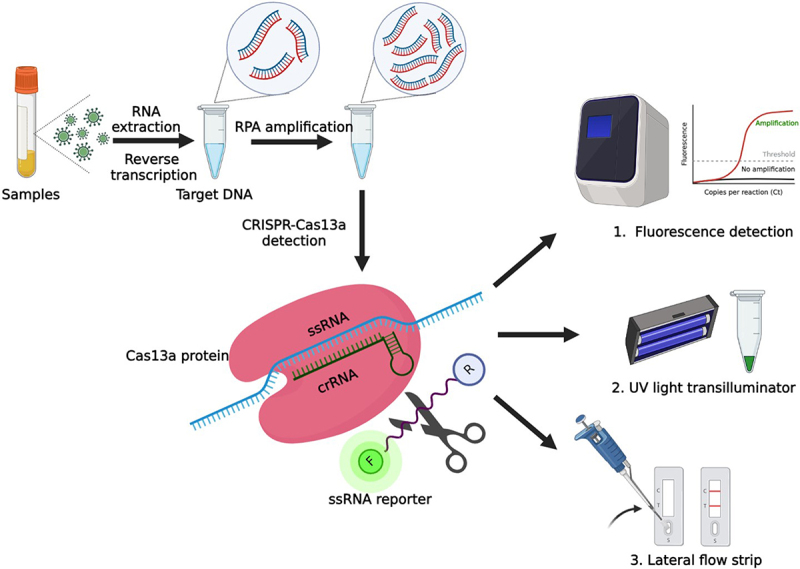
Process illustration of rpa-assisted CRISPR-Cas13a enzymatic molecular diagnostic system.

Our RPA-assisted CRISPR-Cas13a enzymatic molecular diagnostic approach comprises the following four steps: RNA isolation, RPA, collateral cleavage, and signal acquisition (Figure 3). The mechanisms involved contributed to the specific RPA of target sequences, specific binding between crRNA and the target sequence, activation of collateral cleavage ability for Cas13a/crRNA, fragmentation and labelling of DNA, and signal acquisition by sensor system comprising LightCycler, UV, and lateral flow dipstick (Figure 3).

### LwCas13a protein expression and purification

**Figure 4.**
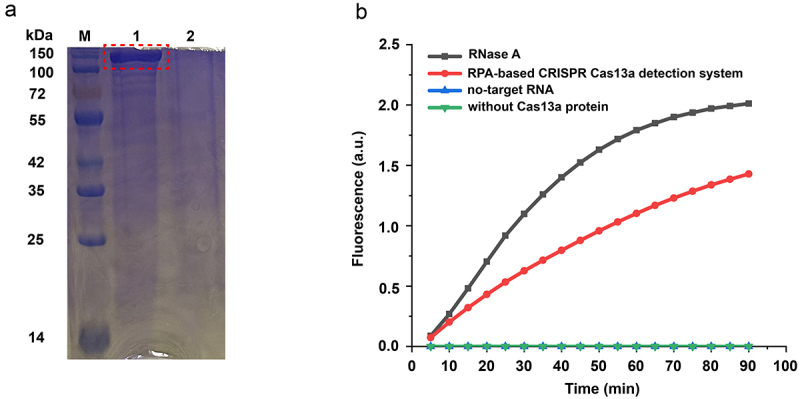
(a) SDS–PAGE gel displaying the molecular mass and purity of LwaCas13a. M, maker; 1, induced SUMO enzyme-treated samples; 2, uninduced SUMO enzyme-treated samples. (b) Activity assessment of purified LwCas13a protein through real-time kinetics for rpa-assisted CRISPR-Cas13a reactions.

The molecular mass of LwCas13a containing SUMO site was 155.2 kDa, whereas the molecular mass of SUMO enzyme-digested LwCas13a was 138.5 kDa. As for LwaCas13a, its molecular mass and purity were revealed by gel staining with Coomassie blue (Figure 4a). The purified protein varying in volume was pooled and buffer exchanged with SB through an ultrafiltration centrifugal tube (Millipore).

### Validation of our RPA-based CRISPR-Cas13a diagnostic system

The crRNA was designed, and transcription was successfully performed. Table 1 lists the target RNA and crRNA sequences. RNA reporters were labelled with 5 “-FAM (fluorescein) fluorophore and 3”-BHQ1. RNase A was used as a positive reference to validate LwCas13a protein activity. As shown in Figure 4b, a fast fluorescence increase was observed for the intact RPA-assisted CRISPR-Cas13a diagnostic system until it reached a peak. In contrast, in the corresponding diagnostic system devoid of target ssRNA or LwCas13a, no reaction was observed. Evidently, the expressed LwCas13a protein was sufficiently active to achieve non-specific RNA cleavage when the target ssRNA and matching crRNA were present.

### Specificity of our RPA-based CRISPR-Cas13a diagnostic system

**Figure 5.**
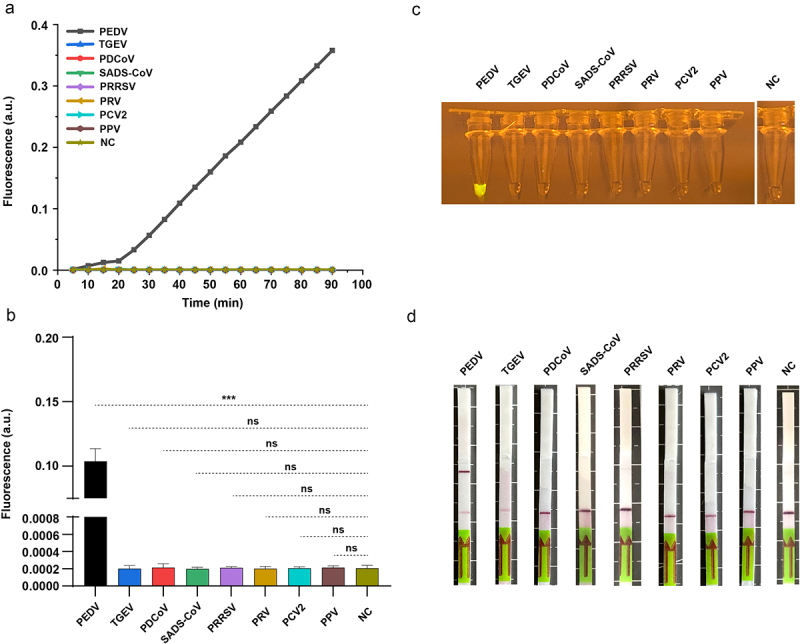
Specificity outcomes for the rpa-assisted CRISPR-Cas13a diagnosis approach utilizing three kinds of sensor systems. (a) Real-time kinetics for the rpa-assisted CRISPR-Cas13a reactions initiated by cDNA samples from TGEV, PDCoV, SADS-CoV, PRRSV, PRV, PCV2, PPV and nuclease-free water (negative control). (b) Final fluorescence value at 40 min (*n* = 3 technical replicates; data presented are means ±sems; ***, *p* < 0.001). (c) Fluorescence visualization of end products under UV light. (d) Lateral flow readout within 1 h.

Under optimized conditions, the RPA-assisted CRISPR-Cas13a diagnostic system was examined for specificity with cDNA from other viruses. The initiation of the sensor system, including the LightCycler and UV, was solely affected by PEDV. However, other viruses, namely TGEV, PDCoV, SADS-CoV, PRRSV, PRV, PCV2, and PPV, did not have any effect (Figure 5a–c). Furthermore, the sensing results with lateral flow strips were consistent with real-time fluorescent readings. As shown in Figure 5d, only the lateral flow dipstick of PEDV exhibited a positive test band, which was absent in the dipsticks of the remaining viruses. Collectively, our RPA-assisted CRISPR-Cas13a diagnostic approach is applicable for the specific detection of PEDV without cross-reaction with additional viruses.

### Sensitivity of our RPA-based CRISPR-Cas13a diagnostic system

**Figure 6.**
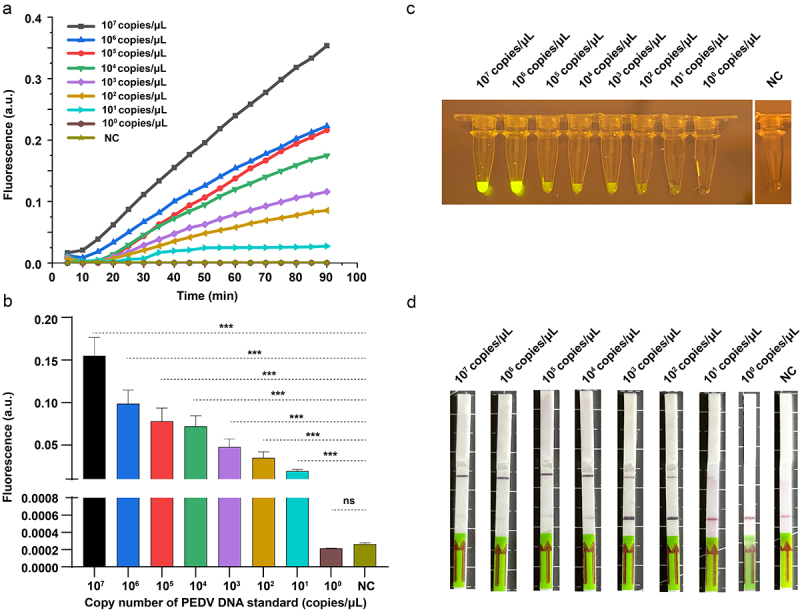
Sensitivity outcomes for the rpa-assisted CRISPR-Cas13a diagnosis approach utilizing three kinds of sensor systems. (a) Real-time kinetics for the rpa-assisted CRISPR-Cas13a reactions evoked via gradient PEDV DNA standard (10^7^–10° copies/µl. (b) Final fluorescence value at 40 min (*n* = 3 technical replicates; results are represented by mean ±sems; ***, *p* < 0.001). (c) Fluorescence visualization of the end products under UV illumination. (d) Lateral flow reading within 1 h.

Under optimized conditions, different concentrations of PEDV DNA (10^7^–10° copies/µL) were used to assess the sensitivity of our RPA-assisted CRISPR-Cas13a diagnostic system. Our RPA-assisted CRISPR-Cas13a diagnostic approach reached 10^1^ copies/µL using the LightCycler and UV sensor systems (Figure 6a–c). Furthermore, the RPA-assisted CRISPR-Cas13a sensing system with a lateral flow dipstick exhibited a sensing limit of 10^2^ copies/µL (Figure 6d), which was consistent with the sensitivity of the corresponding fluorescence assay. Our proposed diagnostic system for PEDV detection showed a limit of 10^1^ copies/µL for all three sensor systems.

### Application in clinical samples

To verify whether our RPA-assisted CRISPR-Cas13a system was consistent with RT – qPCR for PEDV identification, we tested 35 clinical samples. As shown in Table 2, the two approaches yielded identical results for clinical sample detection, revealing 100% concordance between them. Altogether, our results indicate that this RPA-based CRISPR-Cas13a diagnostic system can allow accurate on-site detection of PEDV in a clinical setting without requiring expensive instruments.

**Table 2.** Coincidence rate between the rpa-based CRISPR-Cas13a diagnosis system and rt-qPCR for PEDV detection of clinical samples.

Assay	Number of samples	
	Positive	Negative
Fluorescence detection	29	6
UV light transilluminator	29	6
Lateral flow strip	29	6
RT-qPCR	29	6

## Discussion

PEDV, a significant pathogenic microorganism that induces gastrointestinal symptoms in piglets, results in substantial economic damage to the global swine industry [32]. Owing to the fast transmissibility of PEDV and the absence of efficacious treatment for this viral disease, establishing a fast and precise diagnostic approach to detect PEDV is imperative to achieve rapid disease control. Virus isolation is considered the standard method, but the isolation and identification of PEDV virus is complicated and time-consuming because it is not easily adapted to cell lines and cultured in vitro. While the aforementioned conventional methods have facilitated the development of PEDV detection, the high requirements of instruments and trained personnel have limited their application in the field and in poorly equipped laboratories. To date, a few detection measures for PEDV diagnostics have emerged, including RT – PCR [33], RT – qPCR [34], ELISA [8] and RT-LAMP [35]. However, RT – PCR and RT – qPCR require a thermocycler, and serological assays such as ELISA are strenuous and temperature-dependent, have an increased probability of misdiagnosis, and provide only indirect evidence of PEDV infection. Despite the extensive application of these diagnostic measures for PEDV sensing, they are still not readily implementable in the field because the operating system requires professionalism, and the instruments are costly. Furthermore, Li et al. have formerly described the advantages of RT-LAMP for identifying PEDV in field scenarios or underequipped laboratories [35]. However, there was a main limitation regarding its application for the pathogen identification: because of the carryover pollution attributed especially to the aerosol droplets produced in the course of RT-LAMP assaying, the high assay sensitivity can easily result in false positives. Therefore, a field-deployable PEDV diagnostic system should be developed to overcome early stage disease limitations.

The present study proposes an RPA-assisted CRISPR-Cas13a approach to diagnose PEDV using the M gene of PEDV. Non-professionals can also operate this diagnostic system. Users are required to introduce the necessary reagents into the nucleic acid specimens under test. Then, a reaction occurs inside a thermostatic incubator, and the testing is subsequently completed with lateral flow strips [36]. Pipettes and incubators are the major instruments required for performing the assay, revealing their potential requirement for field-deployable PEDV identification. Our novel nucleic acid recognition approach used impressive sequence recognition of the crRNA complex and promiscuous RNase activity of the succeeding LwCas13a [37]. The former applies to the recognition of gene sequences, whereas the latter applies to readout production using an ssRNA molecule that is doubly labelled by a fluorophore and quencher. Using the designed specific crRNA and RPA primers, CRISPR-Cas13a allowed specific recognition of PEDV, with remarkable sensitivity because of signal amplification via the collateral effect of Cas13a.

In conclusion, our RPA-based CRISPR-Cas13a system for PEDV diagnosis will facilitate the control of PEDV outbreaks in the future. The present findings can improve the biosensing reach based on CRISPR-Cas [38–40]. Regardless of the additional viral detection techniques, the RPA-assisted CRISPR-Cas13a diagnostic system has unique advantages. Along with confirmed reliability, simplicity, sensitivity, specificity, and no requirement for expensive equipment, the proposed approach can be extended and generalized for the identification of other viruses, with promising applicability in a broad spectrum of fields, from disease diagnosis to food safety inspection. Further studies should be performed to simplify the operating procedure using microfluidic chips and other techniques for convenient applications.

## Author contribution

YW performed the experiments, and wrote the original draft; DH designed the project, performed the experiments, analysed the data, and wrote the manuscript; WL analysed the data; YD analysed the data; LF performed experiments; DL analysed the data; YT designed the project; SX designed the project and revised the final version of the manuscript. All authors agree to be accountable for all aspects of the work.

## Ethical statement

The study was approved by The Ethics Committee of Huazhong Agricultural University.

## Supplementary Material

Supporting_Information (6).docx

## Data Availability

The data that support the findings of this study are openly available in https://doi.org/10.6084/m9.figshare.26888605.v1.
