# Supplementary material for: Field-deployable porcine epidemic diarrhea virus diagnostics utilizing CRISPR-Cas13a
Source: Virulence. 2024 Nov 19;15(1):2429022. doi: 10.1080/21505594.2024.2429022 (PMC11581157; doi:10.1080/21505594.2024.2429022)
Supplement: Supporting_Information (6).docx [file KVIR_A_2429022_SM3164.docx]

Supplemental Information

**Virulence**

**Field-deployable Porcine Epidemic Diarrhea Virus** **Diagnostics Utilizing CRISPR-Cas13a**

Yuanyuan Wang^1,3#^, Dalin He^2#^, Weihua Li^3^, Yaqin Dong^3^, Linlin Fang^3^, Deju Liu^3^, Yi Tang^2*^, Shaobo Xiao^1*^

1. College of Veterinary Medicine, Huazhong Agricultural University, Wuhan 430000, Hubei Province, China

2. College of Veterinary Medicine, Shandong Agricultural University, Tai’an 271018, Shandong Province, China

3. China Animal Health and Epidemiology Center, Qingdao 266000, Shandong Province, China

^#^These authors contributed equally to this work.

^*^Corresponding authors: Yi Tang ([tyck288@sdau.edu.cn](mailto:tyck288@sdau.edu.cn)); Shaobo Xiao (wangyy1986@webmail.hzau.edu.cn).

Tel: +86-538-8242027

Table S1 Annealing reaction components

| **Component** | **Volume to add (μL)** |
| --- | --- |
| crRNA template, 100 μM (use sequences in Table) | 1 |
| T7-3G oligonucleotide, 100 μM | 1 |
| Standard *Taq* buffer, 10× | 1 |
| UltraPure water | 7 |

Table S2 In vitro transcription reaction components

| **Component** | **Volume to add (μL)** |
| --- | --- |
| Annealing reaction | 10 |
| NTP buffer mix | 10 |
| T7 RNA polymerase mix | 2 |
| UltraPure water | 17 |
